# Recovery of a Common Bean Landrace (*Phaseolus vulgaris* L.) for Commercial Purposes

**DOI:** 10.3389/fpls.2018.01440

**Published:** 2018-10-25

**Authors:** Cristina Mallor, Miguel Barberán, Joaquín Aibar

**Affiliations:** ^1^Unidad de Hortofruticultura, Centro de Investigación y Tecnología Agroalimentaria de Aragón, IA2 Instituto Agroalimentario de Aragón (CITA – Universidad de Zaragoza), Zaragoza, Spain; ^2^Escuela Politécnica Superior de Huesca, IA2 Instituto Agroalimentario de Aragón (CITA – Universidad de Zaragoza), Zaragoza, Spain

**Keywords:** local varieties, biodiversity, BCMV, germplasm, genebank

## Abstract

The “Caparrona” bean is a landrace that was grown largely in Monzón, and for that reason, it is also known by the name of “Caparrona de Monzón.” Historical references mention that in the thirties of the last century, Caparrona beans reached a production higher than 200,000 kg. Nevertheless, the increasing modernization of agriculture at the end of the 20th century enhanced its replacement by newer varieties. As a result, only a few local growers continued producing Caparrona beans mainly for family use. However, in recent years, the high demand for local products, grown with environmentally friendly farming techniques, has reawakened interest in this local bean. In order to recover the Caparrona bean crop, a study was conducted with the aim of assessing this landrace, along with all the processes, from collecting seeds to securing the *in situ* and *ex situ* conservation. Six bean samples were initially collected from local farmers and the traditional knowledge was also recorded. After the first seed-borne virus test, two samples were rejected because of the positive results for Bean Common Mosaic Virus (BCMV). The four remaining samples were evaluated in a randomized complete block design with three replications at two locations. All through the growth phase of the plants, samples were taken for a virus test. Two samples tested positive for BCMV and were discarded. Between the two healthy seed samples, regarding morphology, chemical composition, and agronomic data, no significant statistical differences were found. Therefore, both samples were selected for commercial production. The seeds obtained from the assays were transferred to a recently created producers’ association, which registered a private label to commercialize the Caparrona beans as a gourmet product. Seeds are also available from the Spanish BGHZ-CITA public genebank.

## Introduction

The vegetable sector plays an important role in the European Union (EU), accounting for 13.7% of EU agricultural output. In 2016, the total production of vegetables in the EU was 63.9 million tons. Spain (24.1%) and Italy (17.4%) were the most important producers (Eurostat Statistic Explained, 2017). In the past, the Spanish vegetable production was characterized by a rich variety of landraces, created by farmers themselves through repeated simple selection procedures, from generation to generation. Unfortunately, this *in situ* biodiversity has been eroded due to intensification of food production and globalization, and, currently, only a few crop varieties are being commercialized, while many local varieties are neglected or underutilized (Barbieri et al., 2014).

However, nowadays, the trend is changing, and many consumers are demanding local food products for economic reasons (increase in farmers’ income, greater added value for local stakeholders, etc.); social benefits (i.e., maintenance of the population in the territory); environmental concerns (decrease in transport and gas emissions, landscape conservation, and biodiversity, etc.); and because local products are perceived fresher or of better quality (Pearson et al., 2011; Richards et al., 2017). The increased consumer demand for diversity in vegetables opens up new avenues for restoring these neglected local varieties (Kreutzmann et al., 2007). In this work, we are interested in a local bean landrace, which was cultivated some years ago, but it is no longer in commercial production.

The common bean is a valuable legume for human consumption worldwide, being an important source of high-quality proteins, carbohydrates, vitamins, minerals, dietary fiber, phytonutrients, and antioxidants (Cardador-Martínez et al., 2002; Reynoso-Camacho et al., 2006). The common bean was introduced into Europe in the early decades of the 16th century from two domestic centers, the Mesoamerican and the Andean (Lioi and Piergiovanni, 2015). The Iberian Peninsula was an expansion zone and a secondary center of diversity for the common bean, generating a wealth of landraces (Santalla et al., 2002).

Among the Spanish common bean landraces, the “Caparrona” bean was grown largely in the locality of Monzón. For that reason, it is also known by the name “Caparrona de Monzón” (Raluy, 1982). Historical references mention that in the thirties of the last century, a great number of farmers produced fruit and vegetables in the Monzón area, in the northeast of Spain, to supply the population of nearby places. The famous local Caparrona beans reached a production higher than 200,000 kg and was commercialized in the Spanish national market (Raluy, 1982). The industrial development meant that most farmers no longer cultivated beans, and only a few local growers continued producing Caparrona beans mainly for family use. However, the high demand for local products, grown with environmentally friendly farming techniques, has reawakened interest in this local bean.

Despite its significance in the past, this landrace is neither cited in the Spanish legume catalog (Carravedo and Mallor, 2008), nor is it represented in the Spanish National Inventory, which includes the passport data of the accessions held *ex situ* in the collections of the public Spanish genebanks. From these aspects, it is evident that the Caparrona beans are currently being threatened by extinction. Thus, there is enough justification for the present study to proceed with the aim of assessing this landrace, which is at high risk of genetic erosion.

To recover the Caparrona bean crop, a study was conducted with the following tasks: (a) to collect samples and obtain traditional knowledge from local orchards; (b) to evaluate the phytosanitary state; (c) to determine differences in morphology and agronomic characteristics and nutritional values; (d) to select the sample with more favorable characteristics for commercial production purposes; (e) to provide good-quality seeds to local farmers for *in situ* production; and (f) to maintain seeds in the vegetable Spanish genebank (BGHZ-CITA) for long-term *ex situ* conservation. Consequently, the aim of this paper is to study the possibility of recovering the “Caparrona” common bean landrace for commercial purposes.

## Materials and Methods

### Sample Collection and Traditional Knowledge

Bean samples were collected from local growers in Monzón (Huesca, Spain). The orchards were visited twice. During the first visit, the plants were in the flowering stage of development and leaf samples were collected to test for seed-borne viral diseases. After assessing the presence or absence of viruses, the seed samples were collected only from the growers of healthy plants, during the second visit at harvest time. These samples were then used to carry out the assays. Additionally, traditional knowledge regarding Caparrona beans was obtained from the growers using a questionnaire in which they were asked for agronomical practices and uses.

### Seed-Borne Virus Monitoring

Leaf samples were monitored for the Bean Common Mosaic Virus (BCMV) and the Bean Common Necrotic Mosaic Virus (BCNMV), because they are the most prevalent seed-transmitted diseases for bean seed production (De Ron, 2015).

To identify the virus, leaf samples were tested serologically by double antibody sandwich-enzyme-linked immunosorbent assay (DAS-ELISA), using specific BCMV and BCNMV polyclonal antibodies from Agdia^®^.

Samples were obtained from mother plants in order to select healthy seeds for the assay, and during the growing season to select healthy seeds for growers. After preliminary analyses in mother plants, accessions showing virus infections were excluded. Finally, four from the six accessions were chosen and used in the study. During the growing season, four DAS-ELISA tests were performed, specifically for samples that were obtained on July 8, July 24, August 26, and October 2, corresponding to 13 days after sowing that corresponded to DAS (first trifoliate fully expanded stage of development), 29 DAS (anthesis), 62 DAS (flowering), and 99 DAS (grain filling).

### Experimental Design and Cultivation Practices

Four bean samples, named CAP01, CAP02, CAP03, and CAP04, were evaluated in a randomized complete block design with three replications at two locations: (1) Monzón (41° 54′ N, 0° 11′ E, 279 m.a.s.l.) with an average temperature from seedling planting to harvest of 21.5°C and total rainfall of 137.5 mm and (2) Montañana (41° 43′ N, 0° 48′ W, 222 m.a.s.l.) with an average temperature of 21.4°C and rainfall of 44.1 mm during the growing season. Each of the three replications or plots consisted of 40 plants that were transplanted into two 20 plant-rows with a row-to-row distance of 1.1 m. apart and within a row distance of 0.15 m, equivalent to a crop density of 6.1 plants m^−2^.

Before planting, the soil was prepared by a rotary cultivator with a roller. The crops were initiated on 9 July, with seedlings cultivated in a greenhouse from 25 June. Plants were drip-irrigated as needed. Harvest was performed by hand at the end of October: in Montañana, 126 days after sowing, and in Monzón, 119 days after sowing. For pest and disease control, plants were sprayed with Abamectin and Spiromesifen for *Tetranychus urticae* and *Trialeurodes vaporariorum* control and Clortalonil and Tiram for *Alternaria* and *Botrytis* control. For weed control, hand weeding was done.

The plants were supported using sticks, in accordance with traditional practice in the geographical area, because of its indeterminate growth habit.

### Evaluation Data

Data related to phenology, yield, and seed characteristics, including quantitative and qualitative traits, were recorded following some of the International Board for Plant Genetic Resources (IBPGR) *Phaseolus vulgaris* descriptor list (International Board for Plant Genetic Resources [IBPGR], 1982). Qualitative traits were estimated for each plot, while quantitative traits were estimated in the detailed number of individuals (seedlings, pods, or grains), as follows:

– Growth habit was determined following the Singh (1982) key for growth habit identification.
– Leaflet length was measured on 10 seedlings, on terminal leaflet of third trifoliate leaf from pulvinus to leaf tip.
– Flowers: color of standard and color of wings were observed in freshly opened flowers.
– Immature pod: length, width, and thickness (in millimeters); weight (in grams); cross-section (very flat, pear shaped, round elliptic, and figure of eight); curvature (straight, slightly curved, curved, and recurving); suture string (stringless, few strings, moderately stringy, and very stringy); and color (dark purple, red, pink, yellow, pale yellow with colored mottling or stripes; persistent green) were determined on 30 immature pods.
– Mature pod: length, width, and thickness (in millimeters); weight (in grams); and number of seeds per pod were determined on 30 mature pods.
– Grain qualitative traits: seed coat patterns (constant mottled, striped, rhomboid spotted, speckled, circular mottling, marginal color pattern, broad striped, bicolor, spotted bicolor, pattern around hilum, and other); brilliance of seed (matt, medium, and shiny); seed shape (round, oval, cuboid, kidney shaped, and truncate fastigiate); and seed colors were observed for each plot.
– Grain quantitative traits: length, width, and thickness (in millimeters) were estimated to calculate length/width and width/thickness relationships, corresponding to more or less rounded shapes or more or less elongated shapes. These parameters were determined on 30 grains.
– Phenology traits on a plot average basis: beginning of flowering (days from sowing until 50% of plants in each plot had at least one open flower); physiological maturity (days from sowing until 90% of plants had dry pods ready for seed harvest); and immature pods at harvest time (number and weight of immature pods) were recorded.
– Agronomic traits: each plot was harvested at physiological maturity stage individually, and the number of total pods, seeds per pod, and the dry weight of seeds were measured to calculate the dry seeds per plant (g/plant); dry seeds per area (kg/ha); the number of pods per plant; and the number of seeds per pod. The 100-seed weight was also calculated as the average of five measurements.

For the nutritional value analysis, the following parameters were determined: the moisture content, in an oven set at 100°C to constant weight; the ash content, by calcination in a furnace at 520°C; the protein content, quantified by the Kjeldahl method and a conversion factor of 6.25; the lipid content, by Soxhlet extraction, using petroleum ether as the extractor; and the carbohydrate content by subtracting the sum of the lipid, protein, moisture, and ash contents from 100 (Association of Official Analytical Chemists, 2005). The calorie value was also calculated in kilojoules (kJ).

### Statistical Analysis

Data was studied by means of ANOVA using the SPSS statistical package. The results were expressed as the means ± standard deviation (SD). The statistical significance of the data was analyzed using univariate analysis of variance (*P* < 0.05), and a *post hoc* Tukey-b test was performed to construct homogeneous groups (One-way ANOVA; SPSS for Windows, version 16.0).

## Results

### Traditional Knowledge

The results of the six interviews showed that the local growers, who continue producing Caparrona beans in the area of Monzón are elderly people, aged from 67 to 78, with an average age of 72 years. The traditional sowing date varied from middle June to the beginning of July, mainly by direct seeding, but also by planting of seedlings. Harvesting takes place from the beginning of November, but local weather heavily influences it. The bean plants are traditionally supported using sticks obtained from the nearest riversides. Flood or blanket irrigation is currently utilized, although some growers expressed interest in changing to the drip system. One of the growers practice organic farming. The Caparrona beans normally are consumed as dry seeds, but another traditional way of consumption is as “granaderas” beans. In that case, the pods are harvested before they dry, the beans are obtained from the immature pods and, unlike dry beans, these “granadera” beans do not need to be soaked before cooking.

### Seed-Borne Virus Monitoring

All the leaf samples obtained from plants in the greenhouse after transplanting and tested on July 8 (13 DAS) showed negative results for both analyzed viruses. The next sampling date was carried out on 29 DAS and the CAP02 plants resulted positive for BCMV virus in both locations. The 62 DAS test carried out resulted positive for BCMV in CAP02 and CAP04 plants. Finally, the last test performed, 99 DAS, corresponding to grain-filling stage, also resulted positive for BCMV in CAP 02 and CAP04 plants. Neither of the tested samples was positive for BCMNV virus. Following these results, CAP02 and CAP04 were discarded.

### Growth Habit and Leaflet Length

**FIGURE 1:**
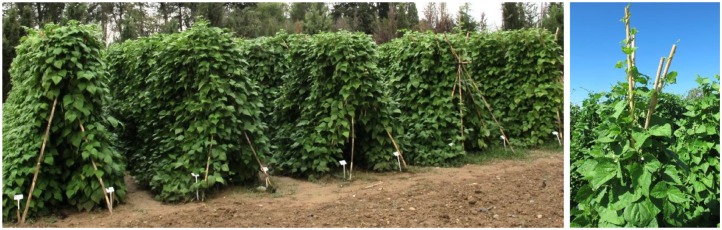
Field trial of Caparrona de Monzón bean.

All the plants presented a type IV climbing growth habit (Figure 1). Although this type of plant usually presents higher seed production, the management is more complicated because of the need for tutoring the plants. Samples were grouped in two clusters according to leaflet length, one formed by CAP01, CAP03, and CAP04 (11.3 ± 1.4 to 11.7 ± 1.3 cm) and the other one formed by CAP02 (10.1 ± 1.9 cm). This size was considered large (>9 cm), regarding the intervals established by Gill et al. (2014).

### Flowers

All flowers presented white with lilac edge standard and white wings. Only one plant from the CAP03 sample presented a plant with flowers completely lilac, including standard and wings. The seeds from this plant were also different (brown seeds); so, it was considered an outside type plant.

### Pods

All immature pods presented pear-shaped cross-section, green color, and were slightly curved, except for the CAP02 sample that presented curved pods (Table 1). No significant differences for quantitative traits related to immature pods were found among the four Caparrona accessions. Regarding mature pods, CAP01 presented higher values, while CAP02 was the lowest, mainly regarding the pod length.

**Table 1:** Mean values for selected pod-related parameters in four Caparrona de Monzón bean accessions (CAP01, CAP02, CAP03, and CAP04) grown in two locations (1: Montañana and 2: Monzón).

	CAP01	CAP02	CAP03	CAP04	*P*
**Inmature pod (location 1)**					
Length (mm)	12.2 ± 0.9	11.8 ± 1.2	12.3 ± 1.2	12.3 ± 1.2	0.387
Width (mm)	15.0 ± 1.2	14.7 ± 1.0	15.1 ± 1.3	15.3 ± 1.2	0.312
Thickness (mm)	7.2 ± 1.7	7.7 ± 1.5	7.3 ± 1.9	7.3 ± 1.9	0.607
Weight (g)	6.6 ± 2.9	8.0 ± 2.2	7.6 ± 2.9	7.6 ± 2.8	0.275
**Mature pod (location 1)**					
Length (mm)	9.5 ± 2.3 a	7.9 ± 1.5 b	9.0 ± 2.4 a	9.0 ± 2.2 a	0.000^∗∗^
Width (mm)	12.2 ± 1.9 a	11.4 ± 1.5 b	11.2 ± 1.6 b	11.4 ± 1.6 b	0.001^∗∗^
Thickness (mm)	9.5 ± 1.7	9.4 ± 1.5	9.3 ± 2.1	9.4 ± 1.9	0.924
**Mature pod (location 2)**					
Length (mm)	11.1 ± 1.6 a	9.6 ± 1.7 c	11.2 ± 1.7 a	10.4 ± 1.6 b	0.000^∗∗^
Width (mm)	10.8 ± 1.3	10.9 ± 1.0	11.0 ± 1.1	10.9 ± 1.2	0.787
Thickness (mm)	10.8 ± 1.5 a	10.0 ± 1.4 b	10.8 ± 1.6 a	10.4 ± 1.4 ab	0.002^∗∗^

*Values are means ± SD; *n* = 30. Means within rows followed by the same letter are not statistically different at 0.05 significance level in Tukey-b *post hoc* test. Significant P-values: ^∗^P < 0.05, ^∗∗^P < 0.01.*

### Phenological Traits

The number of days from sowing to 50% flowering, plants varied between 57 days (CAP01 and CAP04), 62 days (CAP03), and 68 days (CAP04). The harvest was done at the same time in each location. All the samples showed less than 1.5% of immature pods when plants were harvested, except for CAP02 that presented a mean of 5.7% of immature pods at the time of harvesting.

### Grains

**FIGURE 2:**
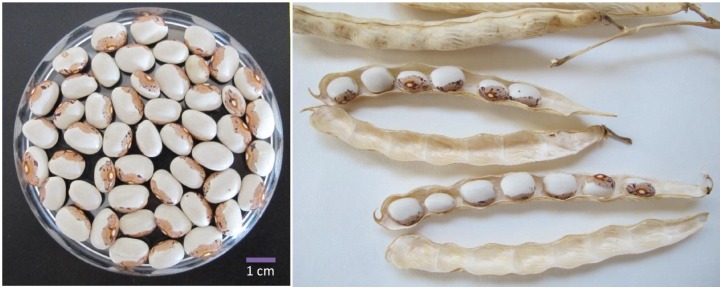
Dry pods and grains of Caparrona de Monzón bean.

All Caparrona samples produced white beans with a brown pattern around the hilum, medium brilliance, and oval shape (Figure 2 and Table 2). Data from original grains, collected from local growers and used for sowing, and grain, which were harvested from both locations, were compared. The length/width ratio was as expected for an oval seed shape in all accessions, with values higher than the unit, ranging from 1.28 (CAP02) to 1.43 (CAP03). On the contrary, the width/thickness ratio also corresponded to an oval cross-section, ranging from 1.12 (CAP02) to 1.40 (CAP01). The lower values of width/thickness grain ratio obtained for CAP02 indicated a shape more rounded, and slightly different to the rest.

**Table 2:** Mean values for selected grain parameters in four Caparrona de Monzón bean accessions (CAP01, CAP02, CAP03, and CAP04) grown in two locations (1: Montañana and 2: Monzón).

	CAP01	CAP02	CAP03	CAP04	*P*
**Grain (original sample)**					
Length/width	1.30 ± 0.01 b	1.28 ± 0.02 b	1.38 ± 0.02 a	1.36 ± 0.02 a	0.000^∗∗^
Width/thickness	1.27 ± 0.07 a	1.12 ± 0.06 c	1.28 ± 0.12 a	1.18 ± 0.10 b	0.000^∗∗^
**Grain (location 1)**					
Length/width	1.35 ± 0.09 bc	1.32 ± 0.08 c	1.43 ± 0.10 a	1.37 ± 0.09 b	0.000^∗∗^
Width/thickness	1.40 ± 0.10 a	1.16 ± 0.09 c	1.32 ± 0.12 b	1.30 ± 0.14 b	0.000^∗∗^
**Grain (location 2)**					
Length/width	1.29 ± 0.08 b	1.28 ± 0.07 b	1.34 ± 0.10 a	1.32 ± 0.07 a	0.000^∗∗^
Width/thickness	1.27 ± 0.08 a	1.12 ± 0.06 c	1.23 ± 0.09 b	1.22 ± 0.08 b	0.000^∗∗^

*Values are means ± SD; n = 30. Means within rows followed by the same letter are not statistically different at 0.05 significance level in Tukey-b post hoc test. Significant P-values: ^∗^P < 0.05, ^∗∗^P < 0.01.*

### Agronomic Traits

Significant differences were found between the two locations regarding agronomic traits (Table 3). The statistical analysis of the yield, estimated by seed yield in kg per ha, showed that this parameter depended on the seed sample (*P* = 0.008) and the location (*P* = 0.000). The interaction between both factors (sample x location) was not significant (*P* = 0.284). In that way, the Montañana plot was more productive than the one in Monzón, due to a better phytosanitary state of the plants. The obtained data showed higher values than the Spanish national mean of 1,899 kg ha^−1^ and similar to the mean values cited in the region of 2,250 kg ha^−1^ (MAPAMA, 2017). According to the classification established by Asensio (2006), this local variety corresponded to a dry bean of high yield and a long life-cycle.

**Table 3:** Mean values for agronomic traits in four Caparrona de Monzón bean accessions (CAP01, CAP02, CAP03, and CAP04) grown in two locations (1: Montañana and 2: Monzón).

	CAP01	CAP02	CAP03	CAP04	*P*
**Location 1**					
Seed yield (kg ha^−1^)	5819.2 ± 163.2 a	4429.3 ± 548.1 b	5762.1 ± 165.7 a	5422.2 ± 29.8 a	0.002^∗∗^
Seed yield (g plant^−1^)	96.9 ± 0.3 a	76.7 ± 9.0 b	96.7 ± 2.7 a	94.2 ± 0.49 a	0.000^∗∗^
Pods per plant	32.9 ± 1.0 a	27.3 ± 3.2 b	31.7 ± 1.6 a	34.0 ± 1.3 a	0.004^∗∗^
Seeds per pod	4.1 ± 0.3 ab	4.6 ± 0.1 a	4.4 ± 0.1 ab	4.0 ± 0.2 b	0.031^∗^
100-Seed weight	71.4 ± 1.0 a	61.0 ± 3.2 b	68.8 ± 1.8 a	69.0 ± 1.3 a	0.001^∗∗^
**Location 2**					
Seed yield (kg ha^−1^)	3508.4 ± 1465.8	3167.9 ± 719.7	4974 ± 1015.3	3698.1 ± 357.7	0.238
Seed yield (g plant^−1^)	33.4 ± 9.9	28.1 ± 4.9	39.0 ± 6.8	30.5 ± 2.4	0.683
Pods per plant	16.9 ± 3.7	17.1 ± 2.1	18.9 ± 5.6	17.0 ± 1.1	0.935
Seeds per pod	3.0 ± 0.5	2.9 ± 0.2	3.5 ± 0.5	2.9 ± 0.2	0.385
100-Seed weight	66.1 ± 3.8 a	56.9 ± 1.6 b	59.7 ± 2.2 ab	61.9 ± 1.9 ab	0.019^∗^

*Values are means ± SD; n = 3. Means within rows followed by the same letter are not statistically different at 0.05 significance level in Tukey-b post hoc test. Significant P-values: ^∗^P < 0.05, ^∗∗^P < 0.01.*

### Nutritional Composition

Of all the studied samples, significant differences (*P* < 0.05) were observed only in moisture and protein contents, with sample CAP04 showing a lower moisture content and a higher protein content, although the differences were not very important (Table 4). Among the most remarkable compounds, common beans are noted for their protein content, being an excellent source of plant-based protein. The total protein content depends on the variety, and ranges from 16 to 33% (Oliveira et al., 2017). The results obtained for Caparrona beans are included in these limits and ranged from 22.1 to 22.7 g.100 g^−1^. Similar values are reported by Rezende et al. (2017) in Brazilian beans (19 to 23 g.100 g^−1^) or by Santalla et al. (1995) in a wide range of samples from Northwestern Spain (22 to 27 g.100 g^−1^).

**Table 4:** Nutritional compositions of four Caparrona de Monzón bean accessions (CAP01, CAP02, CAP03, and CAP04) grown in Monzón (Huesca).

Sample	Moisture (g.100 g^−1^)	Ash (g.100 g^−1^)	Lipid (g.100 g^−1^)	Protein (g.100 g^−1^)	Carbohydrate (g.100 g^−1^)	Caloric value (kJ)
CAP01	7.89 ± 0.08 a	3.97 ± 0.04	1.53 ± 0.18	22.09 ± 0.23 b	64.53 ± 0.54	1529.1 ± 1.6
CAP02	7.83 ± 0.09 a	3.81 ± 0.02	1.66 ± 0.00	22.47 ± 0.03 ab	64.25 ± 0.04	1535.6 ± 1.2
CAP03	8.02 ± 0.07 a	3.93 ± 0.05	1.50 ± 0.01	22.65 ± 0.18 ab	63.91 ± 0.21	1526.8 ± 0.2
CAP04	7.27 ± 0.03 b	4.09 ± 0.11	1.45 ± 0.15	22.70 ± 0.01 a	64.50 ± 0.01	1535.7 ± 5.3
*P*	0.002^∗∗^	0.053	0.414	0.046^∗^	0.263	0.077

*Values are means ± SD; n = 3. Means within columns followed by the same letter are not statistically different at 0.05 significance level in Tukey-b post hoc test. Significant P-values: ^∗^P < 0.05, ^∗∗^P < 0.01.*

## Discussion and Conclusion

The closely related potyviruses, BCMV and BCMNV, are major constraints to common bean (*Phaseolus vulgaris*) production (Worrall et al., 2015). The serological tests showed BCMV virus infection in CAP02 accession in the second sampling date and on CAP04 on the third sampling date. Symptomatic plants were found in the three replications in both locations. Symptoms consisted of mosaic and leaf deformations. Both accessions resulted in being less productive. Accessions CAP01 and CAP03 were virus free in all the tests done. Since the virus is transmitted in a non-persistent manner by aphids and none of the CAP01 and CAP03 plants were infected, this germplasm should be a useful source of genetic diversity for BCMV resistance, although further studies are necessary to confirm the genetic resistance.

Between the two healthy seed samples (CAP01 and CAP03), no significant statistical differences were found regarding morphological, nutritional, phenological and agronomic results. Both samples have been selected for commercial purposes, mainly due to high production and protein content.

Caparrona landrace has been classified as a high yield crop (Asensio, 2006). This fact increases the profit margin per unit area and consequently producers should be encouraged to grow Caparrona beans. Additionally, dry beans are extremely important for human nutrition. Lioi and Piergiovanni (2015) reported that seeds contain from 18 to 28% of proteins, being rich in lysine, which complements the nutritional profile of cereals and tubers. Caparrona beans contain from 22 to 23% of proteins, so it should be considered of nutritional interest for human consumption. The worldwide common bean production has significantly increased in the last three decades, except in Europe, where it has dropped (Lioi and Piergiovanni, 2015). Nevertheless, the recent inclusion of the Mediterranean diet in the UNESCO list of the “Intangible Cultural Heritage of Humanity,” which emphasizes on pulse consumption and the recent proposal of common beans as a nutraceutical food should increase the human common bean consumption.

The obtained seeds from the assays were transferred to a recently created producers’ association called “*Asociación de productores y dinamizadores de la Judía Caparrona de Monzón*,” which registered a private label to commercialize the Caparrona beans. Seeds are also available from the BGHZ-CITA public genebank (FAO code ESP027). The accession is identified by the Genebank code BGHZ5788 and the National Inventory code NC105048.

This study provides a comprehensive model for *ex situ* and *in situ* landrace conservation from collection of local genetic resources to the recovery of Caparrona bean cultivation for commercial production. In that way, this study has allowed to: (1) describe the local bean Caparrona de Monzón using morphological, nutritional, phonological, and yield traits; (2) produce seeds with adequate quantity and quality (virus-free with high-germination rates) for *in situ* conservation by the local growers; and (3) guarantee the *ex situ* conservation in the Spanish public vegetable germplasm bank.

In summary, the local “Caparrona de Monzón” bean has been returned to the fields and now is produced and commercialized, mainly in the local area, as a gourmet product.

## Author Contributions

CM and JA conceived, designed, and wrote the manuscript. All authors contributed to analysis and analyzed the data.

## Conflict of Interest Statement

The authors declare that the research was conducted in the absence of any commercial or financial relationships that could be construed as a potential conflict of interest.
